# Supplementary figures and images for: Ehrlichia Notch signaling induction promotes XIAP stability and inhibits apoptosis
Source: Infect Immun. 2023 Aug 18;91(9):e00002-23. doi: 10.1128/iai.00002-23 (PMC10501217; doi:10.1128/iai.00002-23)

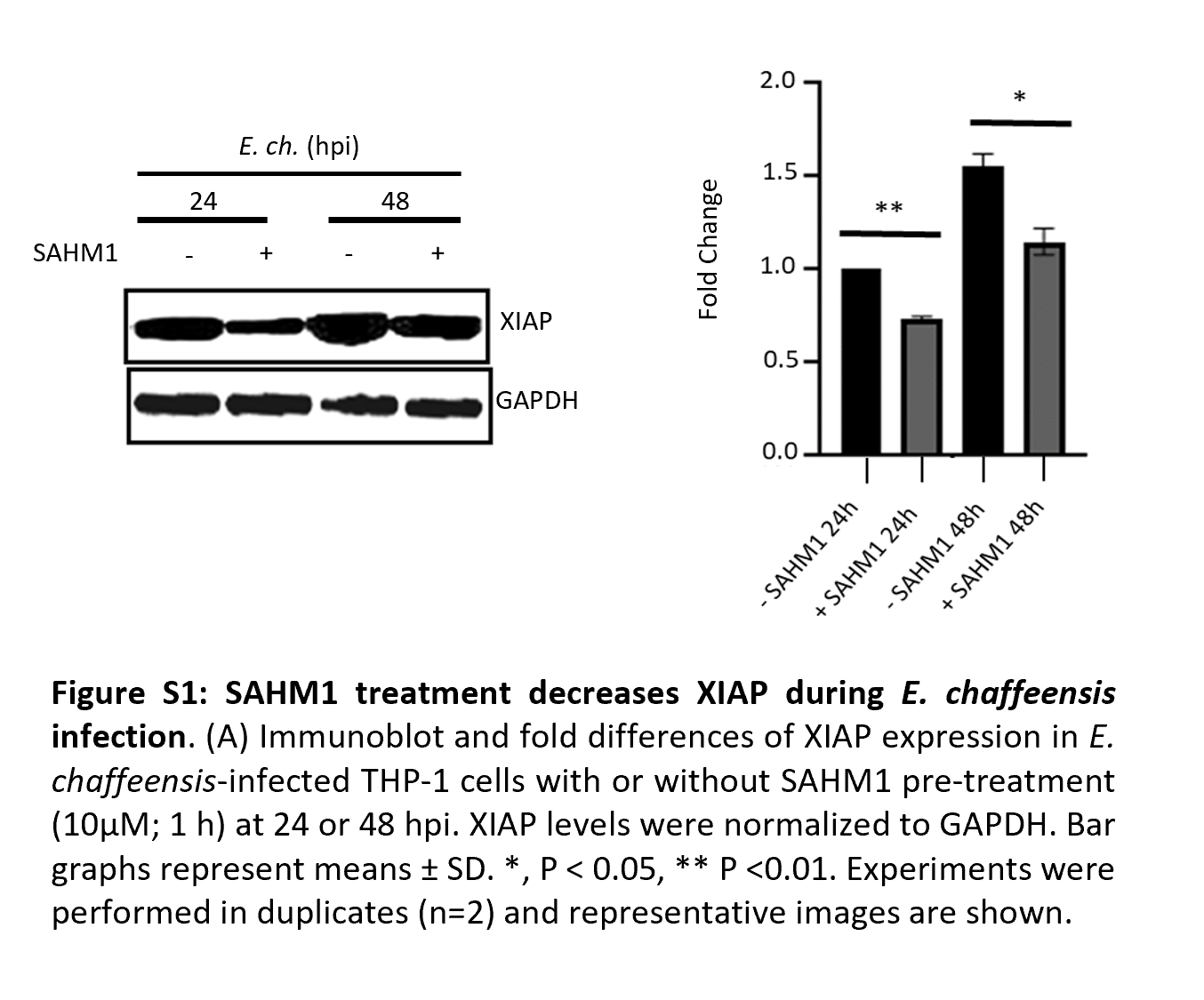

Supplement: Fig. S1 — SAHM1 treatment decreases XIAP during E. chaffeensis infection. [file iai.00002-23-s0001.tif]

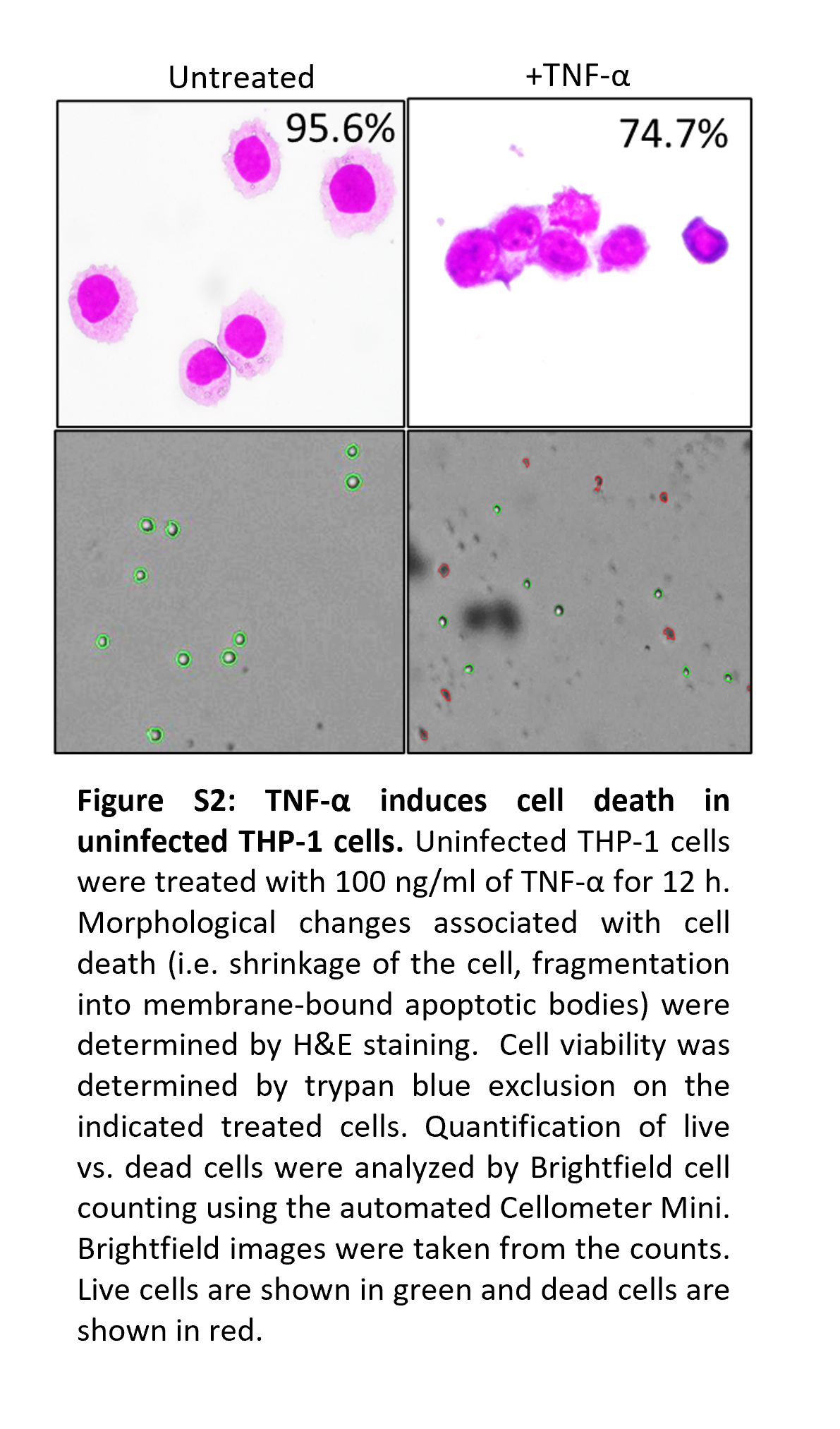

Supplement: Fig. S2 — TNF-α induces cell death in uninfected THP-1 cells. [file iai.00002-23-s0002.tif]

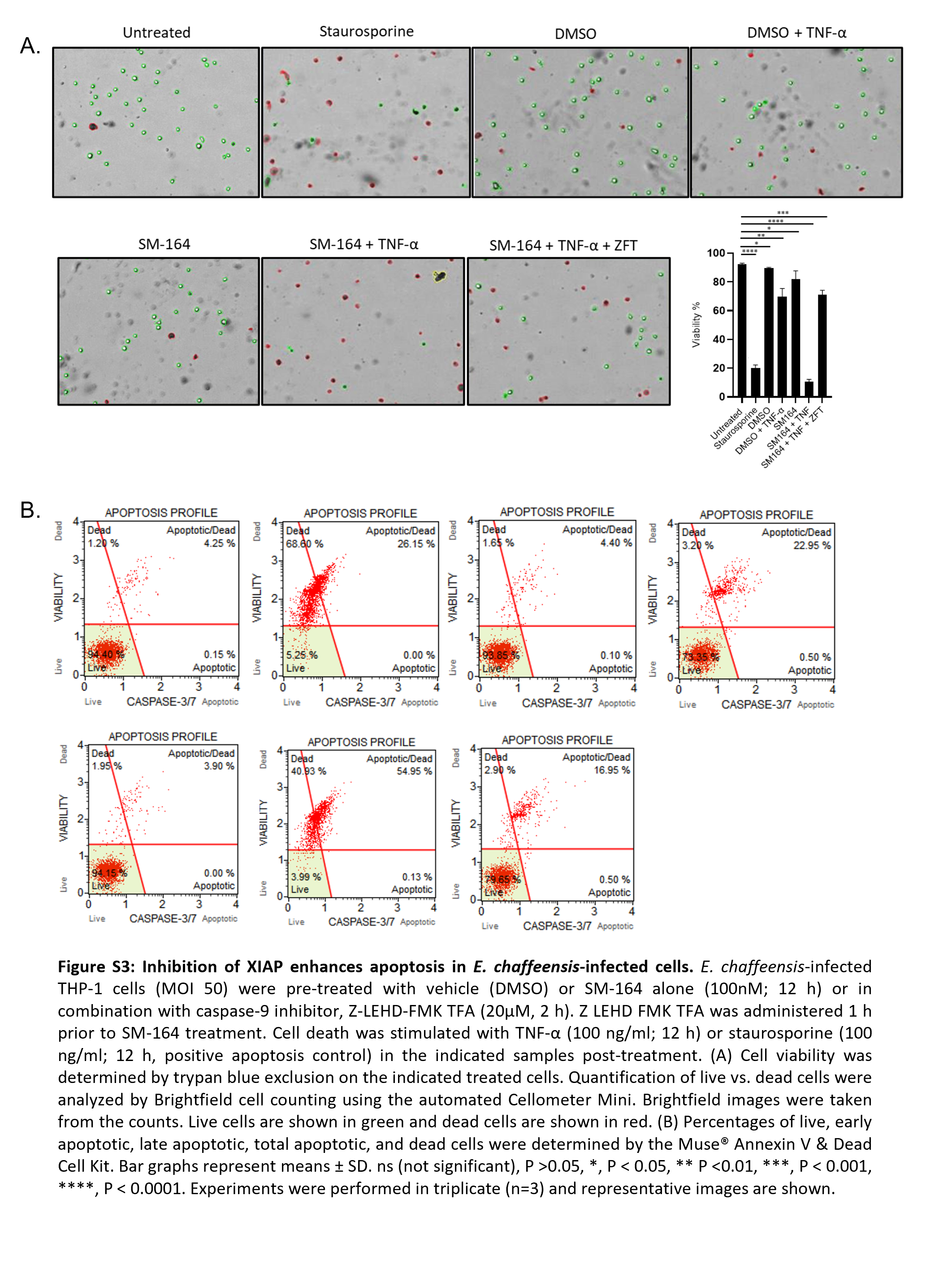

Supplement: Fig. S3 — Inhibition of XIAP enhances apoptosis in E. chaffeensis-infected cells. [file iai.00002-23-s0003.tif]

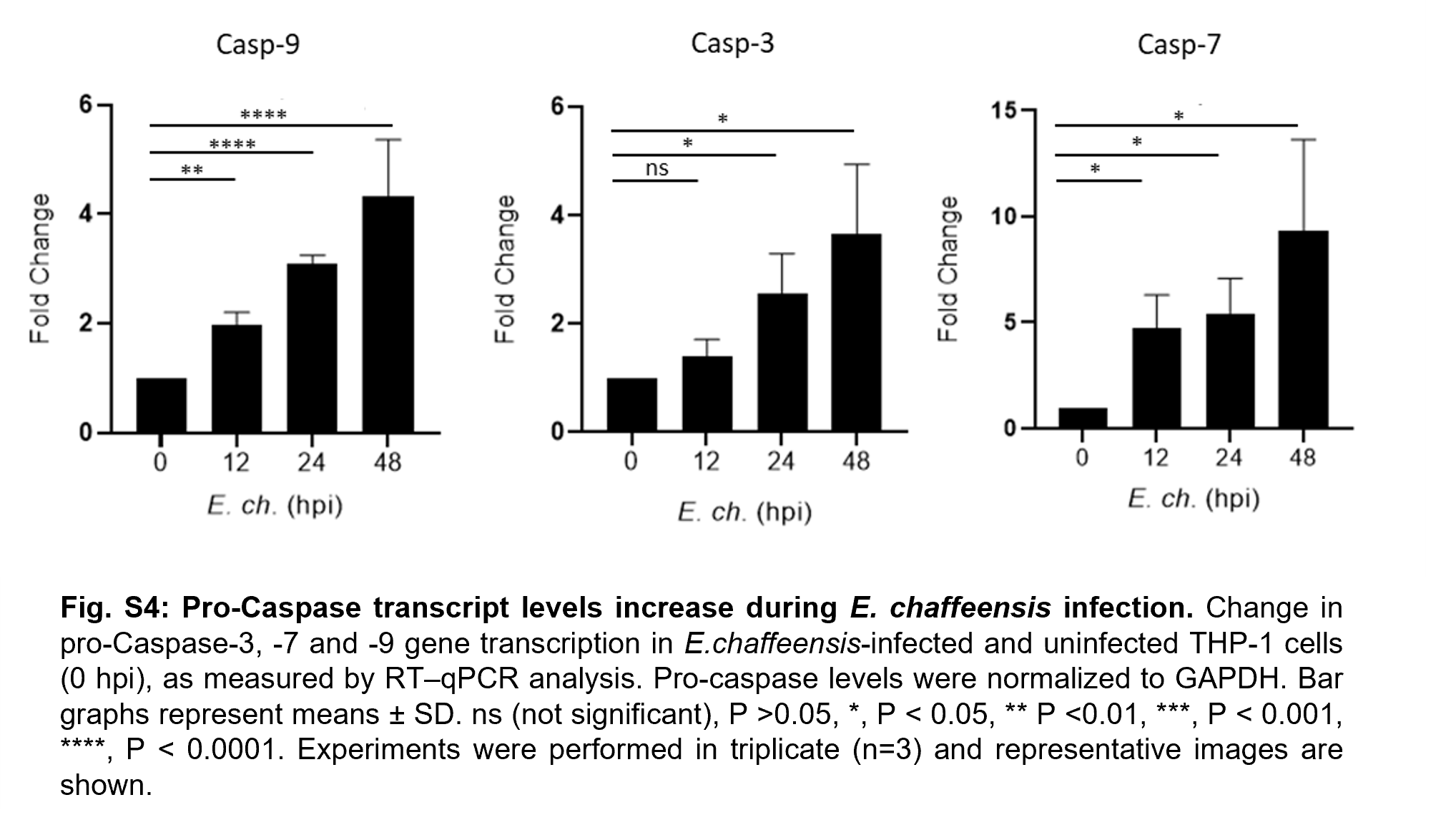

Supplement: Fig. S4 — Pro-Caspase transcript levels increase during E. chaffeensis infection. [file iai.00002-23-s0004.tif]
